# The bioarchaeology of mid-Holocene pastoralist cemeteries west of Lake Turkana, Kenya

**DOI:** 10.1007/s12520-019-00914-4

**Published:** 2019-11-01

**Authors:** Elizabeth A. Sawchuk, Susan Pfeiffer, Carla E. Klehm, Michelle E. Cameron, Austin C. Hill, Anneke Janzen, Katherine M. Grillo, Elisabeth A. Hildebrand

**Affiliations:** 1 Department of Anthropology, Stony Brook University, Stony Brook, NY 11794-4364 USA; 2 Department of Archaeology, Max Planck Institute for the Science of Human History, 07745 Jena, Germany; 3 Department of Anthropology, University of Toronto, Toronto, ON M5S 2S2 Canada; 4 Department of Archaeology, University of Cape Town, Rondebosch, 7701 South Africa; 5 Department of Anthropology and Center for the Advanced Study of Human Paleobiology, George Washington University, Washington, DC USA; 6 Department of Anthropology, University of Colorado-Boulder, Boulder, CO 80309 USA; 7 Department of Anthropology, Dartmouth College, Hanover, NH 03755 USA; 8 Department of Anthropology, University of Florida, Gainesville, FL 32611 USA; 9 Turkana Basin Institute, Nairobi, Kenya

**Keywords:** Pillar sites, Mortuary archaeology, Turkana Basin, Herding, Stone beads, Food production

## Abstract

Early herders in eastern Africa built elaborate megalithic cemeteries ~ 5000 BP overlooking what is now Lake Turkana in northwestern Kenya. At least six ‘pillar sites’ were constructed during a time of rapid change: cattle, sheep, and goats were introduced to the basin as the lake was shrinking at the end of the African Humid Period. Cultural changes at this time include new lithic and ceramic technologies and the earliest monumentality in eastern Africa. Isolated human remains previously excavated from pillar sites east of Lake Turkana seemed to indicate that pillar site platforms were ossuaries for secondary burials. Recent bioarchaeological excavations at four pillar sites west of the lake have now yielded ≥49 individuals, most from primary and some from secondary interments, challenging earlier interpretations. Here we describe the mortuary cavities, and burial contexts, and included items such as adornments from Lothagam North, Lothagam West, Manemanya, and Kalokol pillar sites. In doing so, we reassess previous hypotheses regarding pillar site construction, use, and inter-site variability. We also present the first osteological analyses of skeletons buried at these sites. Although the human remains are fragmentary, they are nevertheless informative about the sex, age, and body size of the deceased and give evidence for health and disease processes. Periosteal moulds of long bone midshafts (*n* = 34 elements) suggest patterns of terrestrial mobility. Pillar site deposits provide important new insights into early herder lifeways in eastern Africa and the impact of the transition to pastoralism on past human populations.

## Introduction

The spread of food production into eastern Africa ~ 5000 years ago coincided with the creation of elaborate megalithic cemeteries, the first of their kind in eastern Africa and some of the earliest monumental architecture known from sub-Saharan Africa (Grillo and Hildebrand 2013; Hildebrand and Grillo 2012; Hildebrand et al. 2018). At least six megalithic ‘pillar sites’ were constructed around paleo-Lake Turkana during a period of major transition. Termination of the African Humid Period (AHP) ~ 5300 years before present (BP) caused lake levels to drop by approximately 55 m, altering deep water fishing habitats and exposing plains that offered new pasture opportunities for herbivores (Garcin et al. 2012; Chritz et al. 2019). Cattle, sheep, and goats were introduced into this emerging niche, ostensibly by herder populations moving out of the drying Sahara (Gifford-Gonzalez 2017; Marshall et al. 1984), marking the first food production in the region. Livestock and herding spread rapidly around Lake Turkana, bringing pastoralist groups into contact with fisher-foragers and changing the cultural landscape. In addition to monumentality, the spread of herding is linked to the emergence of new stone tool and ceramic technologies (Goldstein In Press; Grillo and Hildebrand 2013; Hildebrand et al. 2018), long-distance obsidian trade networks (Ndiema et al. 2011), and evidence for population admixture (Sawchuk 2017). Providing records of both human morphology and cultural behaviour, pillar sites are crucial to understanding how these complex biological and social processes unfolded during this dynamic period.

These mortuary sites have no known analogue within or beyond Africa (Sawchuk et al. 2018). They represent an unusual example of monumentality among mobile herders facing unpredictable circumstances and reflect unique local processes bound up in the spread of food production (Hildebrand et al. 2018). Communal cemeteries may have provided enduring landmarks on a shifting landscape, bringing herders together to form alliances, reify social networks, and exchange resources and information vital for the success of early pastoralism (Hildebrand et al. 2018; Sawchuk et al. 2018). Skeletons excavated from the pillar sites now constitute one of the largest archaeologically derived collections of human remains from eastern Africa, providing evidence relevant to population structure, behaviour, health, diet, and disease among early herders. Analysis of human remains and their mortuary contexts generates novel insights into peoples’ responses to changes in their environment, economy, and society.

Our bioarchaeological analysis focuses on human remains from three pillar sites on the west side of Lake Turkana: Lothagam North (GeJi9), Lothagam West (GeJi10), and Manemanya (GcJh5). The Later Prehistory of West Turkana (LPWT) team investigated these sites between 2009 and 2014, with more limited research at a fourth pillar site, Kalokol (GcJh3). Excavations yielded the remains of at least 49 people, as well as hundreds of isolated human bone and tooth fragments. We present the mortuary contexts for these individuals, including body position, orientation, and burial goods, and we reconstruct sequences that resulted in dense interments within the central platforms of some of the pillar sites. We describe forms of personal adornment found with the burials—primarily stone and ostrich eggshell (OES) beads—as well as rarer examples of ivory, carnivore-tooth, and rodent-tooth items. Although skeletal remains are highly fragmentary as a result of environmental conditions in Turkana and burial within cobble-filled mounds, they are nevertheless informative about age-at-death, probable sex, and community health. Periosteal moulds of long bone midshafts were used to generate cross-sectional geometric (CSG) properties to reconstruct habitual movement patterns. Together, diverse bioarchaeological datasets from the western pillar sites allow us to reconsider previous hypotheses about pillar site construction and use, and, for the first time, explore the lived experiences of the people buried within them.

## Background

**Fig. 1:**
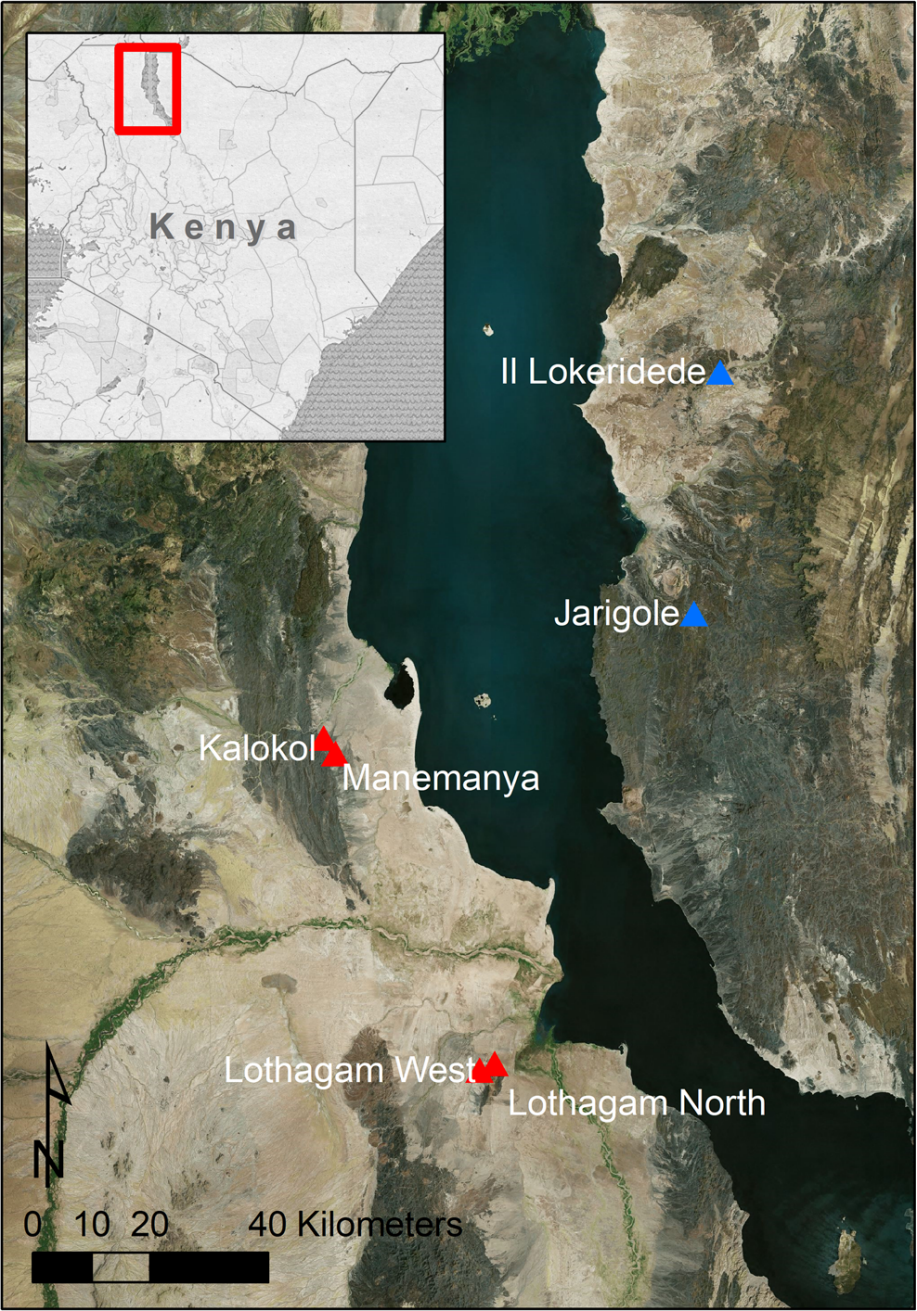
Map of pillar sites around Lake Turkana. Previously excavated sites are in blue; sites described in this paper are in red. Main map © Earthstar Geographics SIO and Microsoft

Pillar sites are named for their linear or semi-circular arrangements of naturally occurring basalt and sandstone ‘pillars’, which people dragged from sources up to 2 km away and placed in circular or elliptical platforms of mounded stony fill. Stone circles and cairns were constructed on the margins of some pillar sites (Grillo and Hildebrand 2013; Hildebrand et al. 2011, 2018; Hildebrand and Grillo 2012). Six pillar sites are known around Lake Turkana: four on the western side, and two on the eastern, all located on imposing points overlooking the paleo-lakeshore (Fig. 1). Today, people living on the west side of Lake Turkana call the sites *namoratunga* or ‘people of stone’ based on a legend that the pillars were human dancers turned to stone by a vengeful deity (Robbins 1972).

Megalithic sites in the basin were first explored archaeologically during the 1968–1970 Royal Geographic Society South Turkana Excavation (Gwynne 1969). The British Institute in Eastern Africa (BIEA) undertook initial excavations at Namoratunga I (also known as Lokori) between 1970 and 1976, documenting individual interments within ‘stone circle graves’ (Soper and Lynch 1977; see also Lynch 1978). These finds led scholars to presume that megalithic sites across the basin would likely contain burials, despite the fact that Lokori differs from most ‘pillar sites’ in having numerous stone circles and no pillars. Namoratunga II, also known as Kalokol (GcJh3) was the first site with pillars to be documented, via surface surveys by Lynch and Robbins (1978, 1979). Subsequent examples of ‘pillar sites’ were reported on the west side of the lake at Lothagam North (GeJi9), Lothagam West (GeJi10), and Manemanya (GcJh5) (Hildebrand et al. 2011; Hildebrand and Grillo 2012), and on the east side of the lake at Jarigole (GbJj1) and Il Lokeridede (GaJi23) (Githinji 1999; Koch 1993, 1994; Nelson 1995). Other potential sites include GbJj4 (Kamau 1991) and an unconfirmed site in the Suguta Valley (Hildebrand et al. 2011).

Jarigole was the first pillar site (sensu stricto*—*having a platform and upright pillars) to be excavated, by the Koobi Fora Field School 1986–1996. Nelson (1995:52)^1^ reported numerous overlapping burial pits in Jarigole’s central mound, with bones and artefacts ‘mixed into the fill as new burials intruded into and scattered the contents of the old’. Lower down, excavators found a primary inhumation of a tighly flexed elderly individual 60–65 cm below the surface of the mound, overlain by large rocks. Other rock clusters were noted but not excavated, raising the possibility that the site might contain deeper primary burials. Excavators did not reach bedrock or sterile deposits before worked at the site ceased in 1996.

Il Lokeridede, ~ 30 km north of Jarigole and ~ 2 km from the habitation site Dongodien (GaJi4), is a smaller pillar site marked by large sandstone slabs (Githinji 1999; Koch 1994; Koch et al. 2002). Limited excavations there also revealed platform mortuary deposits, interpreted as containing secondary burials (see Githinji 1999).

Based on these preliminary findings, Koch (1993, 1994) and Nelson (1995) proposed a Jarigole Mortuary Tradition/Ossuary Complex encompassing all pillar sites within the Turkana Basin. The complex, attributed to early herders, was thought to represent two stages of mortuary activity: a hypothetical primary-burial or other treatment that entailed defleshing, disarticulating, and breaking of human remains, followed by secondary burial of fragmentary bones and grave goods in a communal ossuary. Pillar sites were seen to represent the second stage of activity. Koch and Nelson’s hypotheses—that pillar sites were created by early herders and served as ossuaries for commingled, secondary burial remains—were not resolved by fieldwork at Jarigole and Il Lokeridede. However, their descriptions of the material culture found at these pillar sites, particularly Nderit ceramics, obsidian-based lithic assemblages, and OES/stone beads, established a connection with habitation sites such as Dongodien (Barthelme 1977, 1985) and formed important baselines for the evaluation of other pillar sites.

**Fig. 2:**
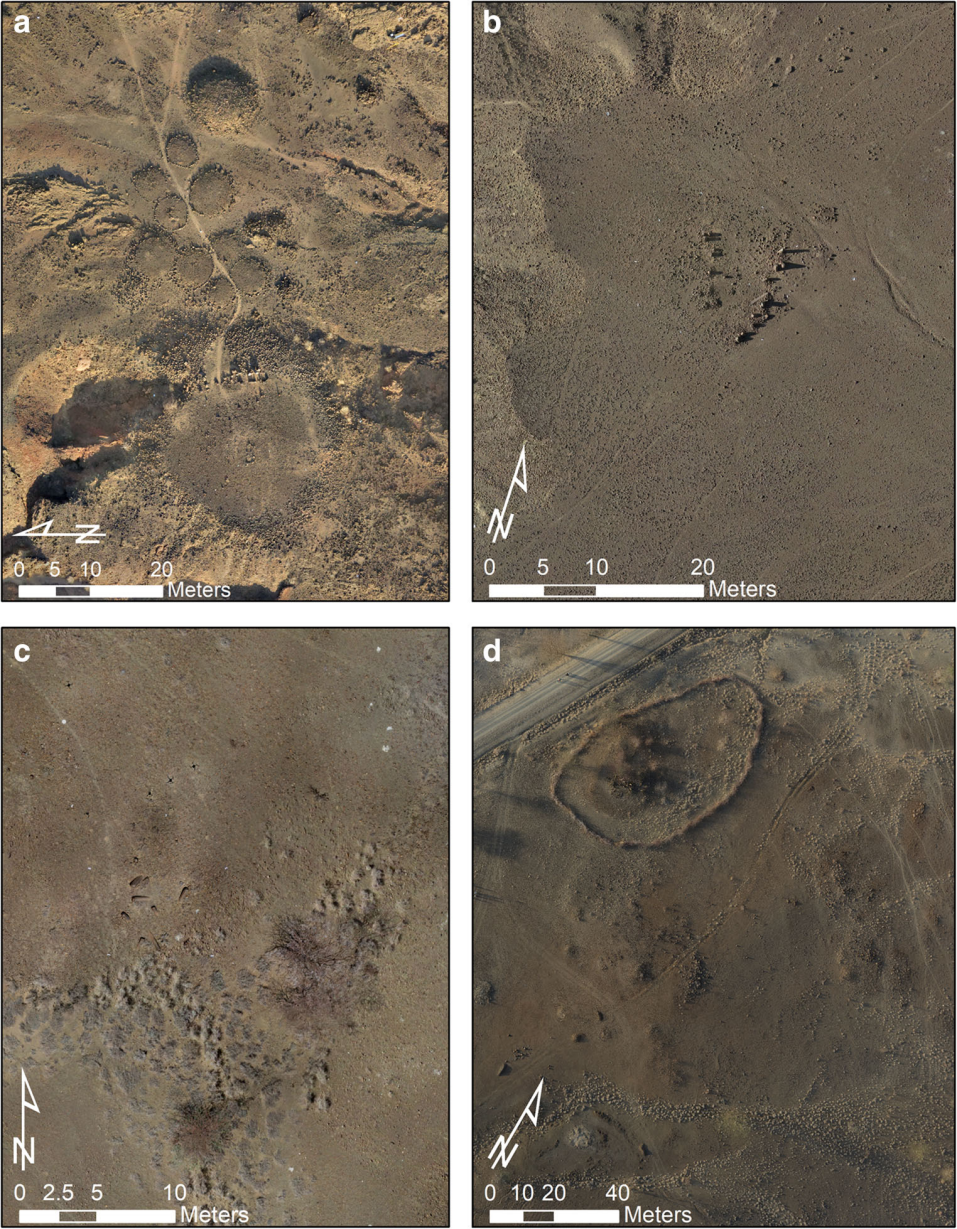
Orthophotographs of **a** Lothagam North, **b** Lothagam West, **c** Manemanya, and **d** Kalokol. All orthophotographs are derived from aerial images recorded via drone and Ground Control Points (GCPs) recorded via total station, processed in Photoscan Pro. Image by A.C. Hill

Between 2009 and 2014, LPWT initiated research at four pillar sites on the west side of the lake: Lothagam North, Lothagam West, Manemanya, and Kalokol (Grillo and Hildebrand 2013; Hildebrand et al. 2011; Hildebrand and Grillo 2012) (Fig. 2). Test excavations at these sites provided radiocarbon dating samples that—combined with dates from Jarigole collections and published age determinations for Il Lokeridede (Koch et al. 2002)—provided the first absolute chronology for pillar sites around the lake. Their chronological overlap with early pastoral habitation sites near Lake Turkana’s paleoshore (4964–4000 cal BP) (Barthelme 1985; Marshall et al. 1984) suggests pillar site construction was coeval with the introduction of herding (Hildebrand and Grillo 2012).

Chronological relations within and between pillar sites raise intriguing points. Three extremely precise dates indicate that Lothagam North, Lothagam West, and Manemanya all saw use within a 43-year period (4868–4825 cal BP), and use of Jarigole during this interval is also likely (Hildebrand and Grillo 2012). Such tight contemporaneity of use lends support for Nelson and Koch’s hypothesis of a basin-wide monumental tradition. Construction and use of pillar sites covered a minimum temporal range of 4853–4541 BP (312 years). The maximum range of use runs from 5270 to 4102 cal BP (1168 years) but includes one late ‘outlier’ date from each of Manemanya, Kalokol, and Lothagam North sites, which might represent subsequent additions or renewal of activities (Hildebrand and Grillo 2012; Hildebrand et al. 2018). Without these outliers, pillar sites’ maximum temporal range runs from 5270 to ~ 4500 cal BP (~ 770 years). Thus, the main expression of this monumental tradition appears to have spanned somewhere between three and seven centuries.

Excavations also revealed inter-site variability in architecture and mortuary and material cultural practices. Although all six sites possess pillars, the number, orientation, and positioning of the stone columns differ (Hildebrand et al. 2011). Associated cairns are found at Lothagam North (*n* = 6), Lothagam West (*n* = 9), and Kalokol (*n* = 2) but not at Manemanya. Lothagam North possesses nine stone-ringed circles in addition. Material culture—notably the presence/absence of Nderit pottery and stone tool raw material preferences—and mortuary patterns vary from site to site. Caprine remains and a zoomorphic bovine palette from Lothagam North (Hildebrand et al. 2018: Fig. 3), together with earlier discoveries of ceramic cattle and sheep figurines from Jarigole (Grillo and Hildebrand 2013: Fig. 3; Nelson 1995), provide additional support for the sites’ architects being herders. However, we do not know the extent to which people engaged in pastoral production (Grillo and Hildebrand 2013; Hildebrand et al. 2018).

**Fig. 3:**
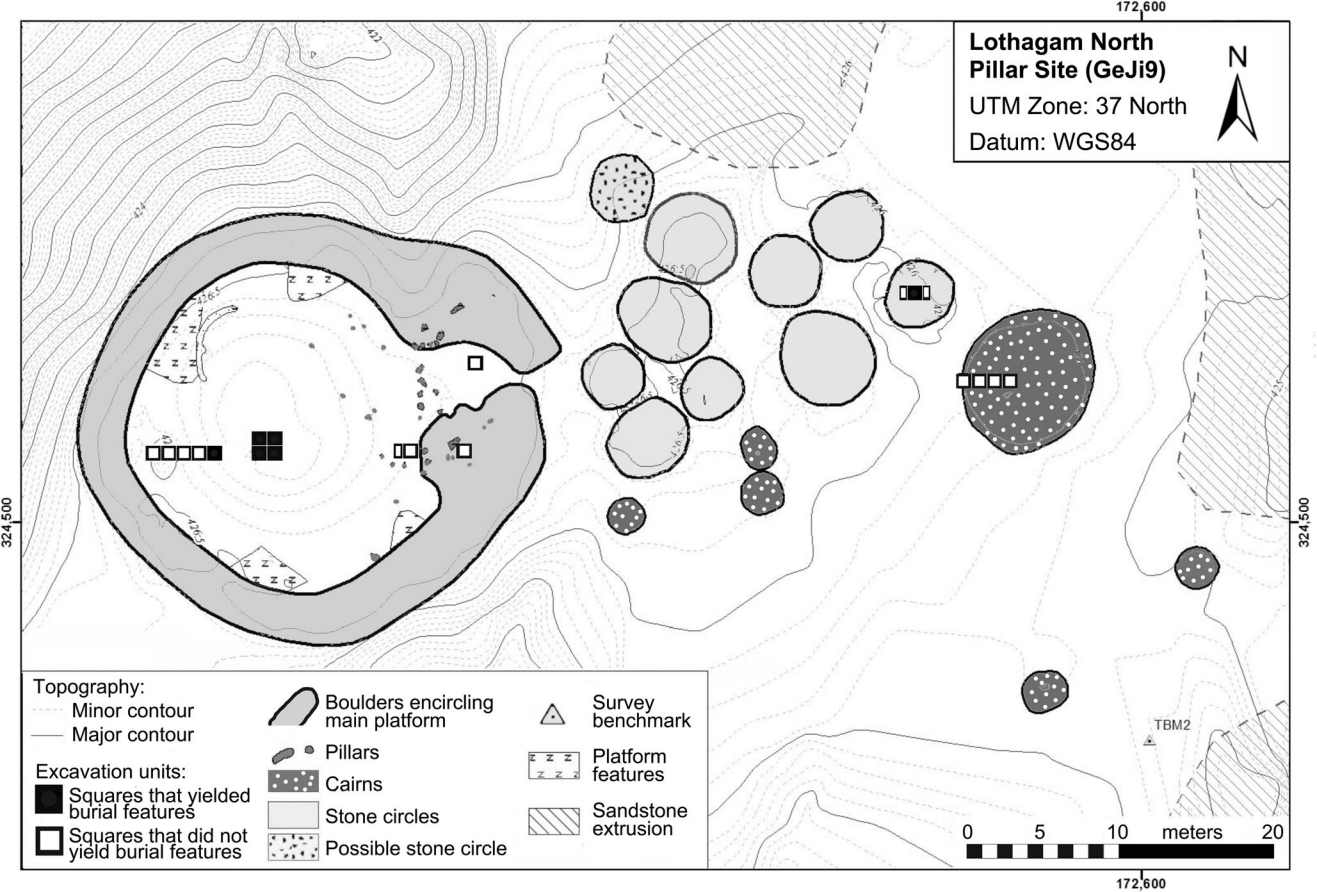
Excavation plan map of Lothagam North indicating areas with human remains (adapted from map by B. Kimeu)

Drawing on new bioarchaeological findings, we can now re-evaluate earlier hypotheses about how pillar sites were constructed and used. For Lothagam North, Lothagam West, and Manemanya, we report funerary practices and associated artefacts. We also explore the biocultural impact of environmental, economic, and social transitions on early herder populations around Turkana as reflected in aspects of human biology. Because excavations at Lothagam North were most extensive, we present results for this site separately from Lothagam West and Manemanya. However, overarching patterns in the mortuary and osteological data provide important information on the people who built these three sites and are pertinent to questions about pillar site function and the degree of cultural homogeneity (Grillo and Hildebrand 2013). We consider whether pillar sites represent a cohesive mortuary tradition, and whether they reflect broadly consistent cultural and burial practices. As little comparative information exists regarding other archaeologically known herding populations within and beyond eastern Africa, this new skeletal collection will be important for future research on the impacts of the transition to pastoralism.

## Methods

**Fig. 4:**
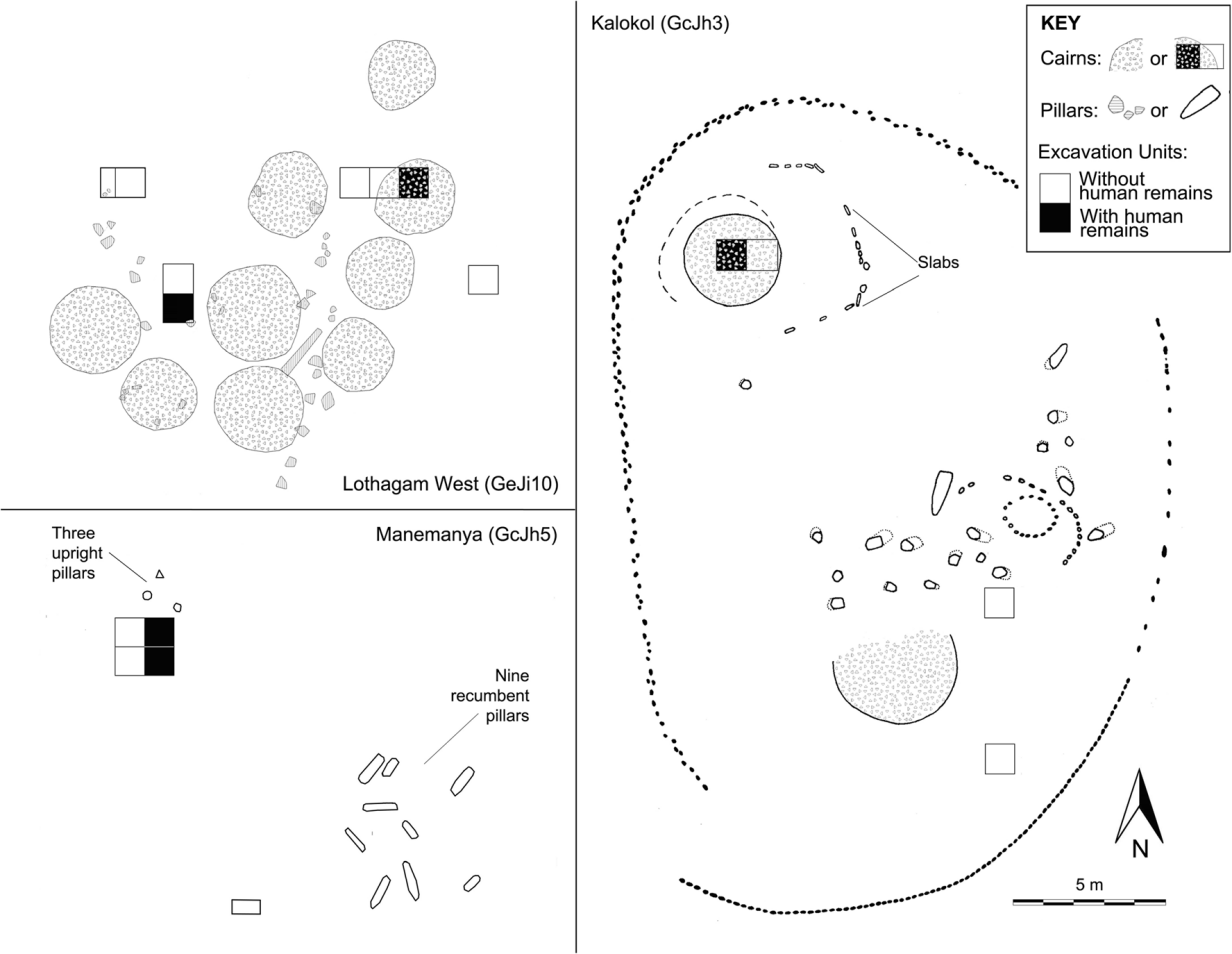
Excavation plan maps of Lothagam West (upper left), Manemanya (lower left), and Kalokol Pillar Site (right) indicating areas with human remains. The Lothagam West map is based on 2012 total station field mapping by B. Kimeu, during which we noticed more and larger cairns than during our brief 2009 observations reported in Hildebrand et al. 2011. The Manemanya map is an updated version of the map presented in Hildebrand et al. 2011:195. The Kalokol Pillar Site map integrates information from maps by Soper (1982:148), Hildebrand et al. (2011:193), and 2012 total station field mapping by B. Kimeu. All maps are to the same scale

Burials were first encountered at Lothagam North and Manemanya in 2009 but were not excavated because a bioarchaeologist was not present at the time (Hildebrand et al. 2011). Bioarchaeological excavations proceeded between 2012 and 2014 and targeted mortuary deposits at Lothagam North, Lothagam West, Manemanya, and Kalokol. At Lothagam North (Fig. 3), a 2 × 2m^2^ unit in the central platform and a 1 × 5m^2^ trench toward its western edge both yielded burial deposits; three other 1 × 1m^2^ units near the eastern edge of the platform did not yield human remains. Among architectural features away from the platform to the east, a 2 × 1m^2^ unit in the centre of one of the stone circles yielded human remains, but 1 × 4m^2^ trench into the side of a large cairn to the east stopped short of the likely location of mortuary deposits. Figure 4 illustrates excavations at Lothagam West, Manemanya, and Kalokol. We targeted three areas at Lothagam West: one 1 × 3m^2^ trench into a cairn on the edge of the platform, one 1 × 2m^2^ unit in the centre platform, and another unit on the north edge of the platform by a cluster of pillars; the first two units yielded human remains. Excavations at Manemanya focused on a 2 × 2m^2^ trench (stepping down to a 1 × 2m^2^ unit) in the centre of the platform that exposed two burials near the base of the platform deposits. A previously excavated unit on the southeast margin of the site did not yield human remains (Hildebrand et al. 2011). At Kalokol, a cairn feature on the northern extent of the site yielded probable human remains that were not excavated. Two previous 1 × 1m^2^ units on the southeast margin of the platform did not yield burial deposits. General excavation and spatial documentation methods for all these sites are described by Hildebrand et al. (2011, 2018).

Bioarchaeological recovery methods combined standard field practices of exposure, mapping, and documentation through images and notes with adjustments developed in response to the intense sunlight and strong winds, complex stratigraphy, and poorly preserved remains. Conditions after interment at all three sites—including poor water drainage, organic sediment, consistently warm-to-hot temperatures, and pressure from the overlying boulder and cobble fill—led to de-mineralisation of bones and tooth roots. Once exposed to dry air and sunlight during excavation, bone would crack and delaminate within minutes. To compensate, excavators worked quickly after initial exposure, kept remains shaded, and stabilized bone as needed with consolidant (Butvar-98, thinned with acetone).

Upon discovery, burials were shaded with UV umbrellas and cleaned with fine brushes to define the limits of the feature and determine completeness, body position and orientation, and associated features. If preservation was fair to moderate, samples for ancient DNA were collected directly from field contexts using gloves, mask, and alcohol-swabbed implements to minimize contamination. We applied consolidant selectively to in situ skeletal fragments in order to preserve their morphology, allowing them to dry while shaded. Consolidant was not applied to small fragments, teeth (mainly crowns, the roots having deteriorated), nor associated artefacts (e.g., adornments).

We recorded the perimeter of each body (where distinct) as well as the margins of discernable burial pits using either a Leica or Nikon total station. We also piece-plotted selected artefacts within the burial fill. In 2013 and 2014, sets of images were recorded for 3D photogrammetric processing at regular intervals as well as at the end of the excavation. Ground Control Points (GCPs) were recorded for photogrammetry processing using the total station and all image sets were processed using Photoscan Pro.

Secondary bundle burials—disarticulated arrangements of predominantly long bones often representing multiple individuals—were documented and removed following a procedure in which each bone was numbered, and photographically documented from a single point a short distance from the unit before removal (Williamson et al. 2003). This permitted subsequent determination of minimum number of individuals (MNI) and osteobiographic analysis after the feature was fully excavated. Given the dense, overlapping nature of burials in the central platform’s mortuary cavity, recovery focused on remains lying entirely within the excavation units. Skeletal elements visible in the walls were documented but not removed.

Artefacts associated with the burials were piece-plotted by total station, then catalogued at the Turkana Basin Institute (TBI) Turkwel facility. Point provenience data were then plotted in ArcGIS. Stone and bone beads associated with specific individuals were photographed in situ, then cross-checked against total station data. Stone and mineral raw materials of the beads and pendants were identified using a combination of visual characteristics, measurement of the specific gravity of a representative sample of the bead artefacts, and archaeometric methods including Raman spectroscopy, XRD, and SEM-EDX (described in Hildebrand et al. 2018 Supplementary Information). This identification includes major classifications (e.g. chalcedony) and sub-categories (e.g. carnelian, or deep-red-to-red chalcedony) as well as reconstructions of the bead manufacture. Ostrich eggshell (OES) beads at Lothagam North number in the thousands and could not always be clearly attributed to individuals but were qualitatively noted within burials. At Lothagam North, OES beads were so numerous that representative samples from certain units/levels were counted to estimate the total number of beads excavated. At Manemanya and Lothagam West, beads were counted fully from all excavation contexts. Although most of the highly fragmentary faunal remains may have been deposited inadvertently as part of the burial fill, several burials included either animal-tooth adornments or near-complete faunal elements that we interpret as mortuary goods. These faunal remains were identified to element and taxon.

After cleaning, reconstruction, and further consolidation (as appropriate, and with tissue set aside for biomolecular analyses), skeletal analysis methods included assessment of age at death, sex, and other osteobiographical details using established methods (AlQahtani et al. 2010; Buikstra and Ubelaker 1994; White et al. 2011). Juvenile ages at death were determined from dental development (AlQahtani et al. 2010) and epiphyseal fusion (White et al. 2011). Adulthood was determined by presence of third molars, epiphyseal fusion, and features like overall bone size, texture, and cortical thickness. Adult age at death was estimated from age-related osteological changes (e.g. at the pubic symphyses, auricular surfaces, and sternal rib-ends, as available) in conjunction with indicators such as degree of dental wear, cranial suture obliteration, and presence of degenerative joint disease (DJD). Wherever possible, adults were grouped into younger (18–30 years), middle aged (30–50 years), and older (50+ years) age categories. Adult sex was determined by pelvic and cranial morphology following standard practice (Buikstra and Ubelaker 1994). Where possible, stature and body mass estimates were based on a combination of in situ long bone measurements, maximum femur head diameter, and maximum metacarpal lengths (per McHenry 1992; Meadows and Jantz 1992; Ruff et al. 1991; Trotter 1970; Trotter and Gleser 1952).

Where preservation permitted, individuals were assessed for pathological changes such as cribra orbitalia, porotic hyperostosis, DJD, dental caries, enamel hypoplastic defects, antemortem tooth loss, periodontitis, and abscessing. Observations were compromised by poor preservation, particularly of cranial material. Limitations arising from poor preservation also affected our capacity to systematically assess skeletal indicators of trauma, disease, and behavioural indicators like presence of squatting facets. Skeletal and dental measurements and non-metric data were collected following standard protocols (Buikstra and Ubelaker 1994; Hillson 2005; Turner et al. 1991). The skeletal collections from Lothagam North, Lothagam West, and Manemanya pillar sites are curated at the TBI-Turkwel facility in Turkana County, Kenya.

Thirty-three adult long bones were sufficiently well preserved to make periosteal casts of estimated mid-shafts for biomechanical analyses. A mould was also made of a femur from the primary burial excavated from Jarigole, curated at the National Museums of Kenya (NMK) in Nairobi. We used Exaflex Heavy Body silicone impression material to create midshaft diaphyseal ring moulds of femora, tibiae, humeri, ulnae, radii, and clavicles. Upper limbs were moulded bilaterally and lower limbs unilaterally, assuming symmetry of lower limb loading (Auerbach and Ruff 2006). Anatomical planes of orientation were marked to preserve correct orientation for digitisation on an Epson flatbed scanner. We estimated the limits of the periosteal boundary by manually tracing along the edge of the digitised moulds using a drawing tablet. Endosteal boundaries were not analysed; however, CSG properties obtained solely from periosteal contours are accurate (Stock and Shaw 2007; Macintosh et al. 2013). Images were analysed using ImageJ platform (Rasband 1997–2012) with the Moment Macrov1.3 plug-in (Ruff 2006) to estimate maximum (*I*_max_) and minimum (*I*_min_) bending strength.

The ratio of maximum to minimum bending strengths (*I*_max_/*I*_min_) reflects diaphyseal shape by comparing two non-fixed axes, indicating a bone’s capacity to resist bending forces (Ruff 2008). Because different activities incur various degrees of long bone shaft bending, comparisons of *I*_max_/*I*_min_ may suggest differences in the types of physical behaviours undertaken in life. The incomplete nature of the skeletal elements precludes analyses that rely on anatomically fixed axes, as well as CSG properties that must be standardized to body size or bone length before conducting comparisons.

Given the dearth of Holocene eastern African comparative data, pillar site individuals were compared to CSG properties from multiple southern African Later Stone Age (LSA) groups: hunter-gatherers inhabiting Mediterranean environments along the Cape Coast, hunter-gatherers involved in some herding in semi-arid areas of the Central Interior, and hunter-gatherers more heavily engaged in herding in arid areas of the Namib Desert. Cape Coast *I*_max_/I_min_ values were obtained using the methodology described above. Data for the Central Interior and Namib Desert southern African groups were obtained using 3D surface scans (Cameron and Stock 2018), results from which are comparable to those obtained with periosteal moulds (Davies et al. 2012; Cameron and Pfeiffer 2014). Humerus, femur, and tibia data were available for all three southern African groups, whereas clavicle, radius, and ulna data were only available for Cape Coast individuals. Comparisons among groups were performed using Kruskal-Wallis ANOVAs, with Mann-Whitney *U* tests for subsequent pairwise comparisons (*α* = 0.05).

## Results

### Mortuary sequence

Pillar sites appear to have functioned as mortuary repositories: all have some combination of primary and/or secondary burials in platforms, stone circles, and/or cairns, and none have evidence for habitation or other activity centres. However, preliminary excavations suggest the scope, density, and architecture of the burial deposits vary among the sites.

#### Lothagam North

**Fig. 5:**
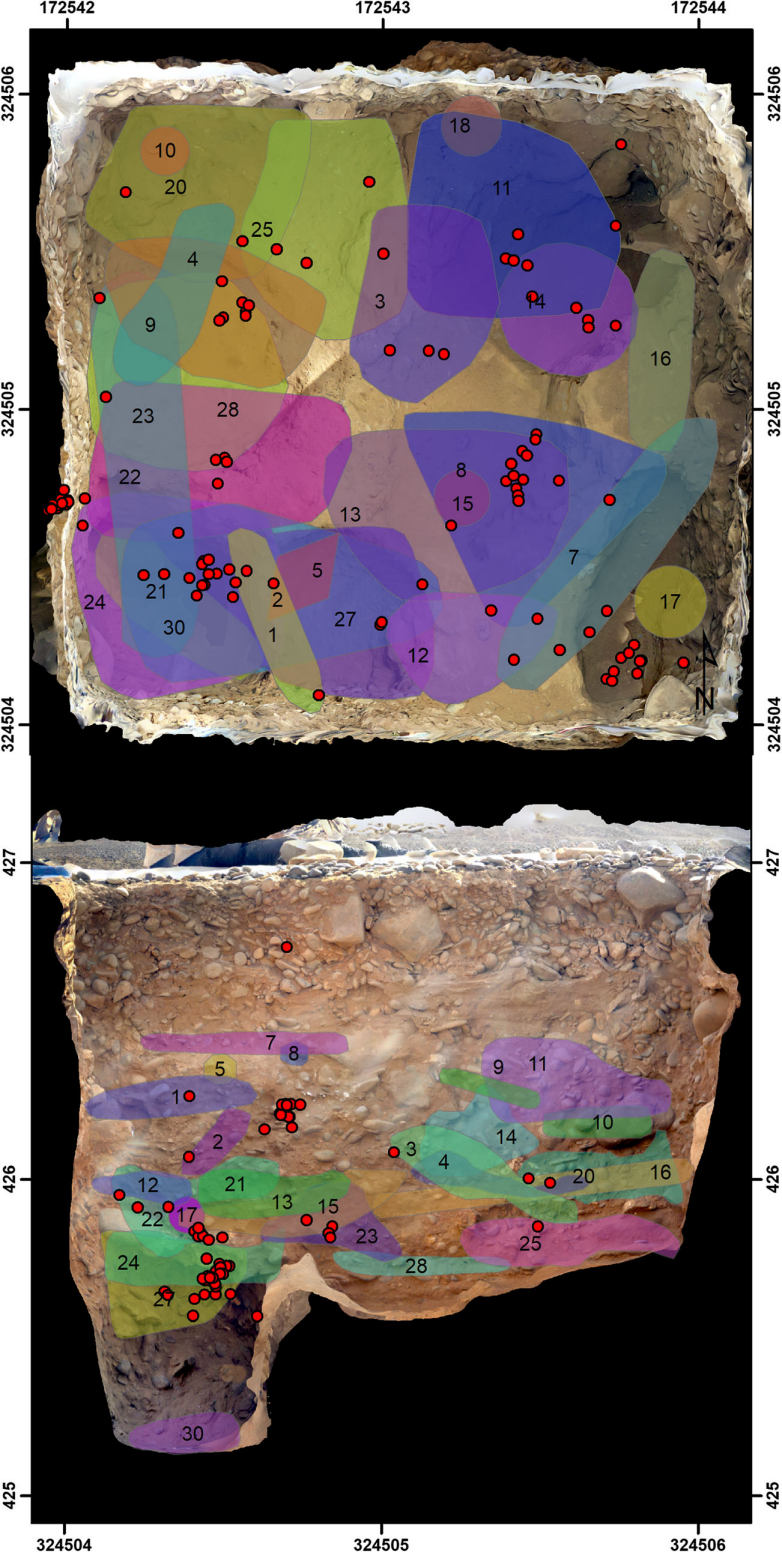
Plan (top) and southern profile (bottom) orthophotographs of unit n04e42 with burial extents (coloured and numbered polygons) and stone beads (red marks). Burial extents are drawn around all point provenience data associated with each skeleton. Stone bead locations were recorded in the field via total station. Note the profile view excludes burials 18 and 19 as these burials are located in a pit along the north wall that is not visible in the southern profile

Extensive excavations at Lothagam North allowed for reconstruction of the burial sequence for deposits concentrated in the central platform (Grillo and Hildebrand 2013; Hildebrand et al. 2018; Hildebrand and Grillo 2012). The site’s construction began with the removal of ~ 120m^2^ of Holocene beach sands to create a deep cavity ringed with boulders and reinforced by sandstone slabs. The first burials were placed in pits dug into the soft sandstone bedrock. After the floor space of the cavity was exhausted, subsequent bodies were added above the pits in various positions and orientations. Based on remote sensing of the mortuary cavity and the number of individuals recovered from a 2 ×2m^2^ unit, the platform contains an estimated minimum of 580 burials (Hildebrand et al. 2018). Positions of the burials documented within this unit illustrate the density of interments within the central platform (Fig. 5).

Platform fill between the burials contained high densities of artifacts, including lithics, ceramic sherds and a ceramic figurine fragment, stone and OES beads, and other adornments. While some artefacts (e.g. many of the stone beads) seem to have been deliberately included as part of a specific burial, others (e.g. highly fragmented pottery sherds that do not refit into whole vessels) appeared to be incorporated into the fill in a more general way (Hildebrand et al. 2018 Supplementary Information). Burial activities stopped before the cavity was completely full, and 30–50 cm of rocky sediment was added, creating a mounded surface capped with rounded, uniformly sized basalt pebbles. Pillars were emplaced within the uppermost cap deposits.

Bodies placed in the central platform did not follow a single funerary convention (Table 1). Two-thirds of the burials were primary; the others were secondary ‘bundle burials’ or features that were too poorly preserved to categorize by burial type. Among primary burials, the bodies were oriented in multiple directions, apparently placed as space permitted: east-west with the head at the east (*n* = 11), north-south (*n* = 6), northeast-southwest (*n* = 5), south-north (*n* = 4), southeast-northwest (*n* = 2), southwest-northeast (*n* = 1), and west-east (*n* = 1). Skeletons with preserved cranial remains were observed to be facing north (*n* = 8), west (*n* = 6), east (*n* = 4), south (*n* = 2), northwest (*n* = 1), northeast (*n* = 1), and southeast (*n* = 1). Individuals tended to be flexed on one side, with arms and legs tightly contracted toward the torso and hands in front of the face. Many individuals were highly flexed to a degree that would have been unnatural in life, suggesting bodies may have been bound, wrapped, or shrouded in this position prior to the onset of *rigour mortis*. Bundle burials were commonly found to be linear arrangements of predominantly long bones representing parts of one or more individuals. We did not observe spatial or funerary patterning with respect to age, sex, or placement of the bundles—men, women, and children who decomposed elsewhere were placed near primary interments. Red ochre staining was occasionally observed on bones within both primary and secondary burials. None of the remains showed evidence of burning or cremation.

**Table 1:** Mortuary context for burials excavated from GeJi9, GeJi10, and GcJh5 (*Ad*, adult, *Juv,* juvenile, *M,* male, *F,* female, *M?,* probable male, *F?*, probable female,*?*, sex undetermined)

Burial	MNI	Burial Type	Age/Sex	Position	Side	Orientation (head)	Facing	Burial context
*LOTHAGAM NORTH (GeJi9)*								
1	1	Primary	Ad/M	Prone, flexed		N-S	E	Boulder on torso, arms and legs flexed beneath body, one hand by face
2	1	Primary	Ad/M	Prone, flexed	R	N-S/SW	W	Directly below B-1, left arm/leg partly extended (slumped)
3	1	Primary	Ad/M?	Flexed	L	E-W	N-NE	Tightly flexed/crouched, slumped onto left side
4	1	Primary	Ad/?	Flexed	?	N-S	E	
5	1	Primary	Infant					Poorly preserved neonate
6	3	Secondary	Ad/M, Ad/?, Juv	Bundle		N-S		Bundle with parts of 3 disarticulated individuals, in pit in centre of stone circle
7	1	Primary	Infant	Flexed	R	NE-SW	SE	
8	1	Primary	Infant	Supine		N-S	Up	Neonate, right arm beneath torso
9	1	Primary	Ad/F	Flexed	R	E-W	N/NW	Left arm flexed around head, face in elbow. Ring of rocks around head, slab on torso
10	1	Primary	Juv	Flexed	L	N-S	E	Slab on torso
11	1	Primary	Ad/F	Flexed	R	NE-SW	N/NW	Slab on head and body, left arm flexed but shoulder high
12	1	Primary	Ad/M	Flexed	R	E-W	N	Slab over pelvis, left arm by chest, right arm semi-flexed under knees
13	1	Secondary	Ad/M?	Bundle		SE-NW		
14	1	Primary	Ad/F?	Prone		E-W	W/NW	Slab over torso, right arm and lower body absent
15	3	Primary	2 Ad/F? Ad/?	Flexed	R/R/?	N-S/N-S/E-W?	*W*/W/S?	Double burial with two flexed adults, third poorly preserved individual near feet
16	2	Primary, Secondary	2 Ad/?			SE-NW (bundle)		Commingled remains of several poorly preserved individuals, at least 1 flexed primary and 1 bundle burial
17	1	Primary	Ad/M?	Flexed	R	NE-SW	NW?	Poorly preserved burial on top of pit
18	2	Primary	2 Ad/M	Flexed	R/R	E-W/NE-SW	N/N	Double burial inside bedrock pit, one on top of the other
19	1	Primary	Juv/F?		L	E-W	S	Poorly preserved burial of ~16 year old in bedrock pit
20	1	Primary	Ad/F?	Flexed	R	E-W	W	Boulder on head, tightly flexed
21	2	Primary	Ad/M? Ad/F?	Prone/Flexed	R	E-W		Flexed, slumped onto stomach. Found with additional isolated right os coxa
22	2	Primary?	Ad/M?Ad/?			N-S/NW-SE		Poorly preserved and commingled remains of at least two individuals
23	1	Primary	Ad/F	Flexed	R	E-W	NW	Lying on bedrock, slabs on head and torso
24	3	Primary	Ad/F, Ad/?, Infant	Flexed	R	NE-SW	W	Primary burial in bedrock pit with parts of second adult and child. Head resting on edge of pit, body curled around possible grindstone
25	1	Primary	Ad/M	Flexed	L	N-S	E	In bedrock pit, slabs on head, shoulders, forearms, and knees
26	4	Primary, Secondary	Ad/M, 2 Ad/M? Ad/?	Flexed, Bundle	R/−	E-W/E-W	N/down	Primary burial with slab on torso, head between two boulders, overlying bundle burial with three disarticulated skulls under boulders and commingled postcrania
27	1	Primary	Ad/?	Flexed	L	NE-SW	S/down	In bedrock pit
28	1	Secondary	Ad/F	Supine		S-N		Secondary burial of an isolated pelvis
29	1	Primary	Ad/M?	Flexed	R	E-W	N/down	In bedrock pit, slab on head and torso
30	1	Primary	Ad/M?	Flexed	R	NW-SE	NE	In bedrock pit
A0	1	Primary	Infant					Head beneath two slabs
*LOTHAGAM WEST (GeJi1)0*								
1	1	?	Juv?					Ribs displaced from an unexcavated pit
2	1	Primary	Juv		R	N-S	W	Child in centre of cairn, skull beneath two large boulders
3	1	Primary	Ad/M	Flexed	L	N-S	E	In bedrock pit, body on steep incline with head low and feet high, covered in cobbles
*MANEMANYA GcJh5*								
1	1	Primary	Ad/F	Partly flexed	R	N-S	NW	Upper body supine, arms extended, legs tightly flexed. Large boulder on skull, body covered with smaller cobbles. Charcoal/ash beneath the body
2	1	Primary?	Ad/?					Articulated feet extending over the torso of Manemanya Burial 1, rest of burial unexcavated

Once placed, primary and secondary burials were covered with rocky sediment that often included a heavy piece of basalt (large cobble or small boulder) or sandstone (typically a tabular slab) on/around the head, torso, and/or pelvis. Several of the crania are warped in a manner that suggests that the resulting pressure was imposed while the bodies were fresh. Over time, bodies were compacted by the overburden of additional interments, and their stone markers as the central platform grew into a mound; some skeletons were compressed to < 5 cm in vertical space. The overburden may also have caused repositioning of some bodies. Among the densely clustered burials of Lothagam North, there are no patterns of differentiation by age, sex, or material wealth as reflected by the presence of personal adornment. One possible example of a dedicated area within the mortuary cavity was the close proximity of three infants and one young child (GeJi9 Burials 5, 7, 8, and 10) found at different depths but all within the southern extent of the 2 × 2m^2^ trench.

One stone circle was excavated at Lothagam North. It held a bundle burial representing at least two adults and one older juvenile. Bone preservation of these elements was significantly better than was seen in the central platform, perhaps because of the shallower depth and lack of rocky overburden. A radiocarbon date on charcoal within this feature falls within the range of dates from the central platform (Hildebrand et al. 2018), suggesting either contemporaneity of use or later recycling of platform fill to a new context.

#### Other pillar sites

Excavations at Lothagam West and Manemanya were less extensive, but it is clear that these platforms also contain multiple burials. Two partial individuals were recovered from pits within the central platform at Lothagam West, with additional pit features visible in profile. At Manemanya, the lower 1 × 2m^2^ step of excavations into the platform encountered the feet of an arthritic adult jutting perpendicularly over a burial of a young adult woman. Their positioning suggests the presence of diversely oriented burials, similar to Lothagam North. Platform deposits at both sites were shallower than at Lothagam North, and we encountered sterile substrates beneath the lowest burials. At Kalokol, no human remains were encountered during 2009 excavations into the platform, although our units were placed along the southeast periphery which may have been outside the extent of burial deposits (see Fig. 4).

Cairns at Lothagam West and Kalokol contained burials, but these interments may post-date creation of the central platforms at their respective sites. At Lothagam West, a ~ 5-year-old child was recovered from the centre of a cairn just east of the central platform on the opposite side of the line of pillars. Excavation of the cairn’s margins revealed it to have been dug into platform deposits. At Kalokol, excavations of one cairn in 2012 revealed a central pit containing probable human remains which were not excavated; this feature also intruded into pre-existing platform deposits. In 2017, local collaborators reported looting of a different cairn located just west of the site, approximately 20 m away from the platform outside the curb. Fragmentary human remains were salvaged, but the degree of sun-bleaching and weathering indicated they had been exposed for some time. The poorly preserved bones belong to at least one adult; age and sex could not be determined. The antiquity of the cairns at Kalokol and their association with the platform and pillars remain unclear.

### Cultural materials from the burials

#### Lothagam North

Ornaments, primarily stone and OES beads, were the most common artefacts found in direct association with burials. We recovered 302 stone beads/pendants from deposits at Lothagam North, of which 173 were directly associated with 20 of the 31 excavated burial features (Table 2, Fig. 5). Other burials likely also contained beads, but the proximity of bodies and rocky matrix made it difficult to associate items with specific skeletons. Stone beads span a range of raw materials, colours, and forms (Hildebrand et al. 2018). Mineral and rock groups include (in descending order of frequency) zeolite (analcime), amazonite, talc schist (soapstone), chalcedony, volcanic rock (typically andesite, phonolite, or basalt host rock), fluorite, and limestone. Individual beads made from calcite, gypsum, chlorite (schist), haematite, and an iron precipitate were also noted. Analcime (45%) and amazonite (32%) dominate the assemblage, making for a primarily pink-and-blue colouring. Colours range from orange/red (chalcedonies), deep to light purple (fluorites), dark green (talc), off-white (limestone), and shades of black (volcanics); a brilliant white scolecite (zeolite) bead and a haematite-cemented sandstone pendent add lustre to the collection. Many of these stones and minerals are also represented in the collections from Jarigole, including amazonite (Nelson 1995).

**Table 2:** Ornamentation and other inclusions for burials excavated from GeJi9, GeJi10, and GcJh5

Burial	Stone bead (*n*)	Stone bead materials	Ornament description	Other inclusions
Lothagam North GeJi9				
1	1	Volcanic (basalt)	Stone bead at neck	
2	5	Amazonite	Stone earring at left temporal, stone beads at neck	
3	5	Talc, chalcedony (carnelian), scolecite	Gerbil-tooth headpiece, stone earring, stone beads near knees, ivory bangles on left arm	
4	24	Amazonite, analcime, limestone (stromatolite)	Stone beads and earring, hyrax teeth lying on chest	
5			OES beads at wrist	
6				Turtle vertebra
7	2	Amazonite	Stone beads at neck, OES at pelvis	
8	1	Amazonite	Stone bead at right arm	
9	5	Amazonite, talc	Stone bead necklace	
10	1		Stone and OES beads at neck, OES at pelvis	
11	8	Amazonite, volcanic (basalt), fluorite	Stone and OES beads at neck	
12	12	Amazonite, volcanic (basalt), analcime, limestone (stromatolite)	Stone beads at neck, stone earring near forearm, ivory at chest, bone beads at hip and left ankle	Ochre staining on skull
13				Ochre-stained rock
14	1	Limestone (stromatolite)	Beads and earring at neck, ivory squares at chest	
15	19	Analcime	Stone beads around two individuals’ necks, ivory bangle around one wrist	Ochre staining on skull
16			Bone and OES beads, ivory squares, and possible tusk	
17			None recovered	
18			None recovered	
19	33	Amazonite, analcime, ivory	Stone bead necklace	
20	1	Amazonite	Stone and OES beads within burial fill	
21			OES beads within burial fill	Ochre staining on arms, torso, cranium
22			Four ivory rings on one left hand, beads by right hand	
23	1	Analcime	Stone bead necklace	
24	2	Amazonite, analcime, ivory	Stone bead necklace, OES beads near pelvis, stone and OES beads within burial fill	Possible grindstone
25	1	Talc	Stone bead near mandible	
26	29	Amazonite, analcime, ivory	Twelve perforated hippo tusks, stone and bone bead necklace with primary burial; stone bead necklace, earring, loose stone beads, and ivory ring within bundle burial	Zoomorphic stone palette (between 26 and 29)
27	2	Fluorite, volcanic (basalt)	Stone beads and ivory within burial fill, OES beads near left shoulder	
28			None recovered	
29	1	Amazonite	Stone bead within burial fill	Zoomorphic stone palette (between 26 and 29)
30			None recovered	
A0			OES beads within burial fill	
Lothagam West GeJi10				
1	1			
2	2			
3	3		OES beads within burial fill	
Manemanya GcJh5				
1	329	Rhyolite, calcite, sandstone	Abundant stone and OES beads covering body, perforated carnivore teeth pendants and bracelet, ivory bangles	Ivory and modified bone object found near body
2				

**Fig. 6:**
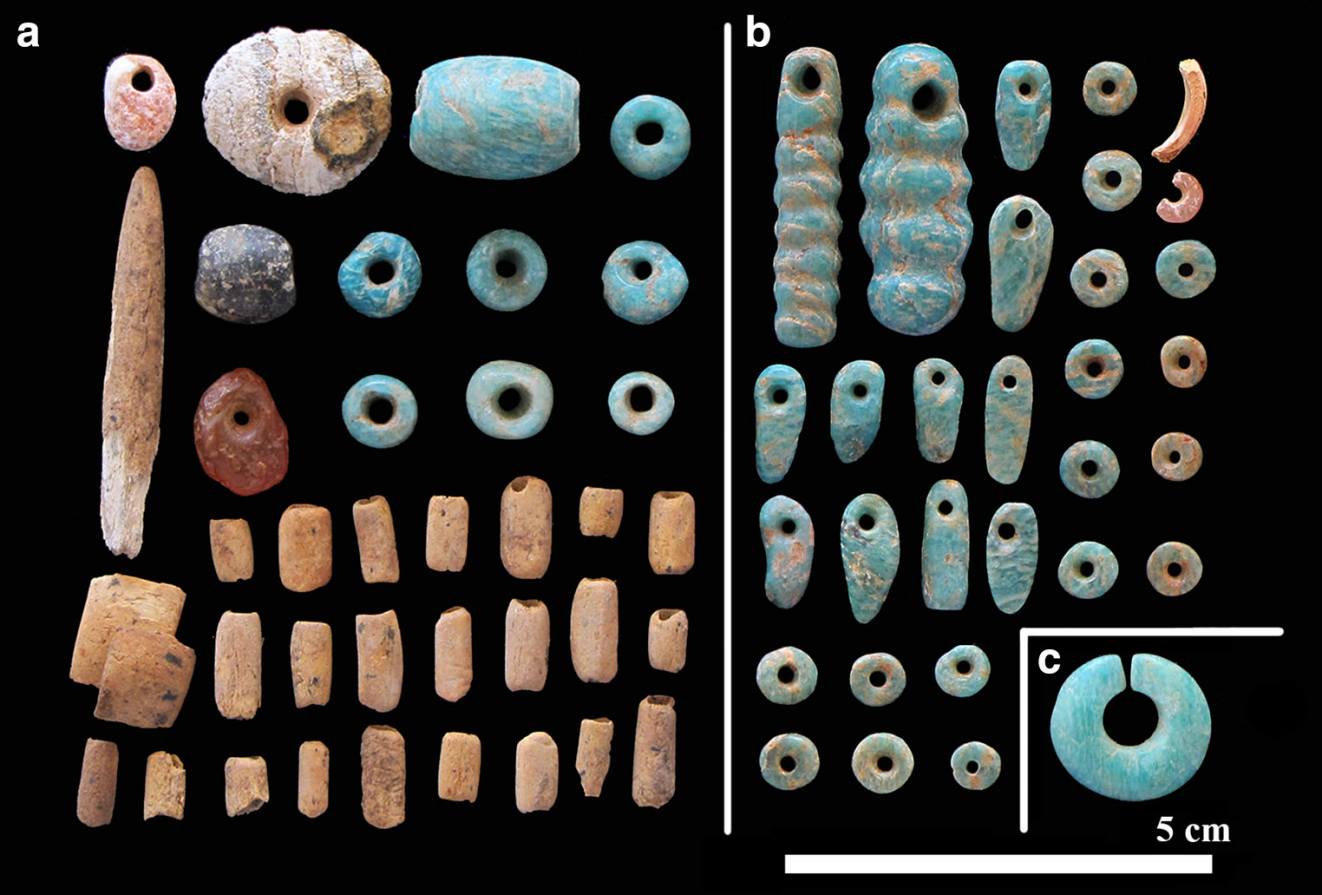
Examples of stone beads accompanying the burials within Lothagam North’s central platform, including **a** GeJi9 B-12’s assemblage of analcime, stromatolite, amazonite, carnelian, and talc (soapstone) beads, found in conjunction with a bone point; **b** GeJi9 B-19’s assemblage of primarily amazonite beads and pendants as well as a broken analcime bead and a fragment of rodent tooth/ivory; and **c** a large amazonite ‘earring’ found with GeJi9 B-29. Image by Carla Klehm

Most were shaped into round beads of varying sizes; other forms included pendants, and incomplete circles that may have been designed as earrings. Jewellery observed in situ includes single ornaments (e.g. an amazonite ‘earring’ with GeJi9 B-29), groupings manufactured from a single type of mineral (e.g. a necklace made of amazonite beads and pendants with GeJi9 B-19), and groupings that included various combinations of raw materials, colours, and forms (e.g. amazonite, basalt, analcime and stromatolite stone beads and organic beads with GeJi9 B-12) (Fig. 6). Mineralogical surveys of the region suggest that many of the raw materials, such as the zeolite and chalcedony, could have been sourced close to Lothagam North. Other materials such as the fluorite and amazonite could have come from the Rift Valley, but from more distant and more distinct (i.e. unitary) sources. Bone beads made from fragile, small faunal diaphyses were also found (GeJi9 B-12, see Fig. 6). OES beads were ubiquitous throughout the central platform deposits. Bead counts from a representative subset of four units (of the excavated ten) yielded 522 whole and 37 partial OES beads, suggesting a total of 1250–1500 OES beads for the Lothagam North excavated units.

Other grave goods from Lothagam North include faunal elements; some apparently incorporated into garments. Around the occipital portion of the skull of GeJi9 B-3, 405 gerbil (*Gerbiliscus* sp.) incisors representing at least 113 animals were configured in a bricklike lattice pattern, suggesting they had been fixed to a headpiece or wrap whose supporting material did not preserve. GeJi9 B-4’s torso was found with 49 hyrax (cf. *Heterohyrax* sp.) upper incisors from at least 25 animals. Ivory ornaments and bangles were found with several of the burials. GeJi9 B-26A was buried next to 12 perforated hippo (*Hippopotamus amphibious*) tusks that may have been strung together and worn. This feature overlaid a bundle burial containing three skulls facing into the ground as well as commingled post-cranial bones (GeJi9 B-26B/C/D). East of the platform, an unmodified Nile softshell turtle (*Trionyx triunguis*) vertebra was found within the stone circle bundle burial (GeJi9 B-6). These examples stand apart from other faunal remains recovered from burial fill, which were highly fragmentary and generally could only be identified to mammal, bird, or fish categories.

#### Other sites

**Fig. 7:**
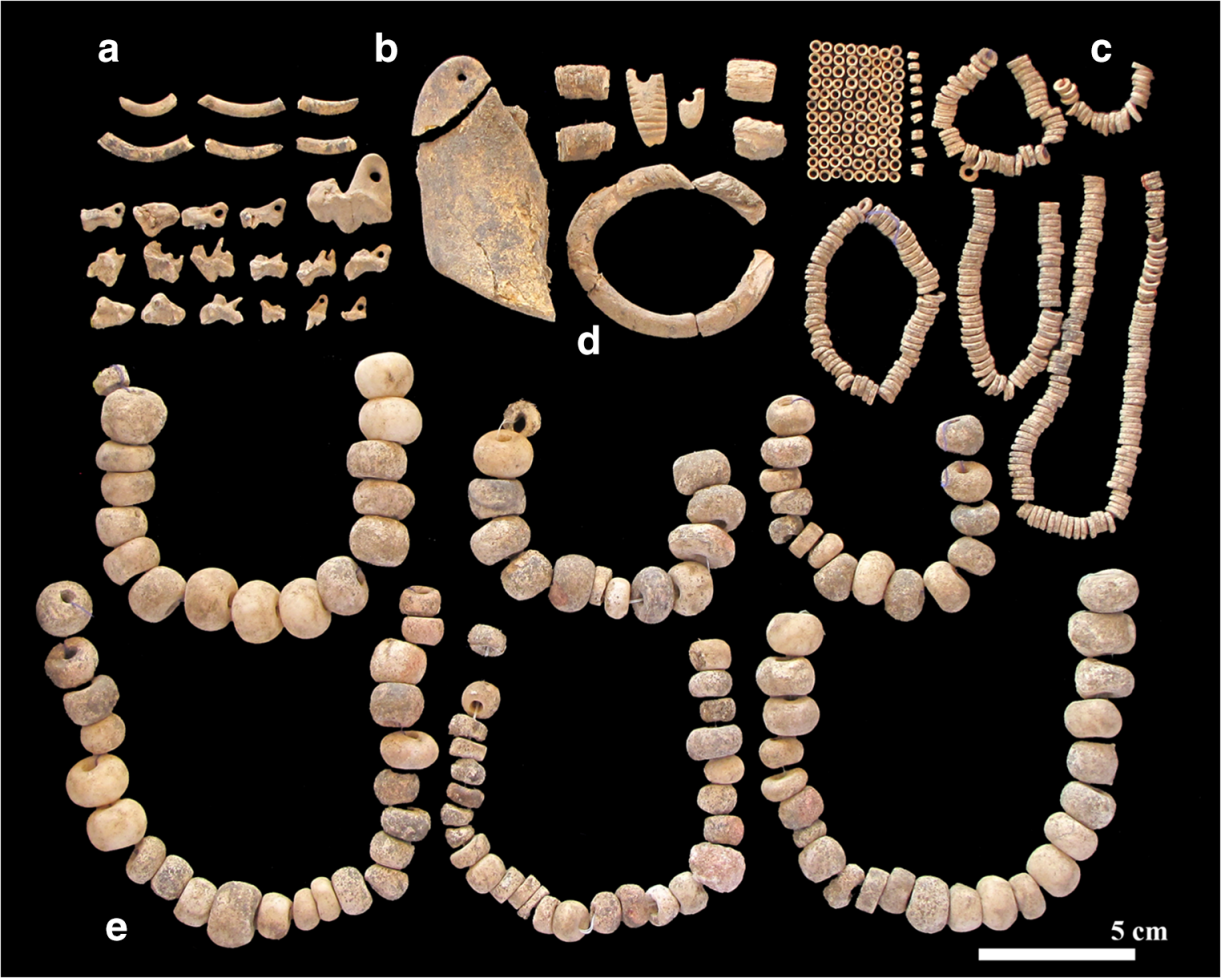
Examples of stone, shell, and bone adornments found with GcJh5 B-1 at Manemanya. These include (A) ivory fragments, *Hyaena hyaena* pendant, and *Canis* sp. tooth bracelet beads; (B) an unidentified bone and ivory object; (C) large and small ostrich eggshell beads; (D) an ivory bracelet, pendant, and other fragments; and (E) numerous large calcite stone beads strung into necklaces, many of which were found still in situ and strung in order (as pictured here). Image by Carla Klehm

Only one complete burial was excavated from Manemanya, but the feature included extensive cultural material (Fig. 7). The torso of a probable woman in her late 20s to early 30s (GcJh5 B-1) was covered by 329 stone beads of somewhat diverse shapes and sizes, further accompanied by at least 9559 whole and 978 broken OES beads. Beads were densely concentrated on and immediately around her body, suggesting they were contained with the body within a wrap or shroud. She was buried on her back with her arms at her sides and legs tightly flexed to the right. The body appeared to have been lain on a relatively flat, undisturbed surface. There was charcoal beneath the body. She wore a necklace that included five gourd-shaped stone beads. Hundreds of larger, round stone beads encircled her torso and pelvis in linear arrangements, suggesting they were part of a garment. Twenty-six exceptionally small OES beads clustered bilaterally near her ears, probably worn as elaborate earrings, while staggered dyads of slightly larger OES beads likely decorated a garment or placket extending down her legs. Numerous strands of OES beads were draped over her rib cage, spine, and pelvis but did not extend beneath her torso, nor did they go around her neck. In addition to beads, she wore perforated carnivore teeth as ornaments—pendants on her upper torso made from lion (*Panthera leo*) and hyena (*Hyaena hyaena*) teeth, and a bracelet of 42 canid (*Canis* sp.) teeth around her left wrist (Fig. 7). An ivory bangle encircled her right wrist. An unidentified, badly degraded composite object made of worked bone and ivory was found near her right shoulder. The perforation of the bone component suggests it was worn or hung, and the size of its bone and ivory components are consistent with a very large animal, possibly hippo.

As noted, we encountered a pair of feet from a second individual above GcJh5 B-1’s torso and observed human bone in the walls of the 1 × 2m^2^ lower trench, indicating other burials are present in deeper reaches of the platform. Further excavation would be needed to determine if the degree of ornamentation seen with the young woman was typical, or if this individual represents an unusual case.

The stone beads at Manemanya are similar to one another in size and shape (as seen in Fig. 7). They were fashioned from raw materials different from those used at Lothagam North. They are almost exclusively calcite, with one bead made from sandstone. With colour variations only slight (off-white to soft pink), the Manemanya beads lack the dramatic hues of the Lothagam North assemblage, but their larger dimensions would still have strong visual impact. Their materials would have been readily available and are easier to work than some of the harder materials (e.g. chalcedonies) found at Lothagam North. The seemingly simpler material technology of the GcJh5 B-1 assemblage, however, is contrasted by the number of accompanying beads. This one burial yielded a stone bead count equivalent to 30 burials at Lothagam North, and more than five times the estimated number of ostrich eggshell beads from Lothagam North’s larger mortuary excavation area.

The other two excavated pillar sites, Kalokol and Lothagam West, yielded fewer burials and, unsurprisingly, almost no burial goods. Only ~ 10 OES beads total were recovered from Lothagam West, most of which were in association with the most complete burial GeJi10 B-3. No beads were recovered from Kalokol.

### The human remains

Skeletons excavated from Lothagam North, Lothagam West, and Manemanya represent both males and females, and range in age from neonates to the elderly (Table 3, *n* = 49). Of the 39 adults and one older adolescent in this sample, 18 are probable males and 12 are probable females. Ten individuals are too poorly preserved or incomplete to estimate sex. Of the individuals whose ages at death could be estimated, eight are young adults (~ 18–30 years), 10 are middle-aged (~ 30–50 years), and five appear to be elderly (50+). Sixteen could only be assessed as adult. Five infants (newborn to 2 years) and five juveniles (2 to 18 years) were also recovered. The scarcity of complete long bones limited estimations of stature, mass, and proportionality. Based on a small sample of adults, males’ stature averaged around 169 cm (*n* = 9) and females were around 163 cm (*n* = 3) (methods per Meadows and Jantz 1992; Trotter 1970; Trotter and Gleser 1952). Average body mass estimated from femur head size is approximately 62 kg (*n* = 3, all probable males) (McHenry 1992; Ruff et al. 1991). Coefficients of variation (CV) for metric variables with a sample size of ≥ 10 are relatively low (mean = 13.28%, *n* = 20 variables) suggesting that the people buried at these sites may constitute a deme—a subgroup of closely related individuals (Table 4).

**Table 3:** Estimated age/sex of excavated individuals (*n* = 49)

Site	Probable M	Probable F	Juvenile/undetermined	Birth-2 years	Juvenile 2–18	Young adult (18–30)	Middle adult (30–50)	Older adult (50+)	Adult (18–50+)
GeJi9	17	11	16	5	3	7	10	4	15
GeJi10	1	0	2	0	2	0	0	1	0
GcJh5	0	1	1	0	0	1	0	0	1

**Table 4:** Statistical summary of measurements (all in mm) for which ≥10 adults could be assessed

Bone	Variable	Adults (*n*)	Mean	S.D.	C.V.
Mandible	Condyle (ant-pos)	12	9.27	1.16	12.56
	Condyle (med-lat)	12	19.86	2.47	12.46
Clavicle	Midshaft (ant-pos)	14	11.30	1.47	13.03
	Midshaft (sup-inf)	14	9.91	1.58	15.89
	Circumference	11	38.32	3.14	8.20
Humerus	Midshaft (max)	11	19.87	1.94	9.76
	Midshaft (min)	11	17.38	2.49	14.33
Capitate	Length	11	20.87	2.96	14.18
	Width	10	19.20	2.81	14.61
	Depth	10	13.41	3.28	24.47
Hamate	Length	10	20.86	2.06	9.89
	Width	11	18.08	2.91	16.08
	Depth	11	16.60	5.49	33.06
Lunate	Length	10	16.29	1.82	11.19
	Width	10	14.89	1.83	12.28
3rd metacarpal	Midshaft (med-lat)	10	8.60	0.70	8.20
	Midshaft (dors-palm)	10	7.91	0.66	8.37
	Base (dors-palm)	10	14.58	1.53	10.48
Patella	Length	10	37.74	3.04	8.05
	Depth	12	19.22	1.64	8.51
Average					13.28

### Indicators of health

We noted no indications of endemic disease or violent causes of death. The most commonly observed pathological features are osteoarthritis, dental hypoplasias, and healed fractures. Osteoarthritis (OA) and degenerative joint disease (DJD), characterized by articular cartilage destruction and associated bony porosity and lipping at joints, are relatively common among the adults. Twenty of the 39 adults have preserved joint surfaces that could be scored for OA, of which 14 skeletons (70%) show some combination of osteophytes, lipping, and/or eburnation. These were typically observed in the spine, shoulders, knees, and feet, although differential preservation of skeletons prevents systematic scoring of all joint surfaces. OA is found more commonly among the middle-aged adults (5 of 8 observed, 63%) and older adults (3 of 4 observed, 75%) compared to the young adults. Distribution is relatively equal between the sexes.

Among the dentitions, two of the 25 individuals possess caries (*n* = 3 of 331 teeth that could be assessed), and all cases are slight. Tooth wear is moderate: the average mandibular third molar wear score is 2.4 (*n* = 14), where cusps are moderately blunted with only pinpoint dentine exposure, if any (Smith 1984). Dental calculus is pervasive, particularly in older adults. Calculus is present on most teeth that could be scored (66%, *n* = 426 teeth assessed). The only case of abscessing was observed in GeJi9 B-25 on the left maxilla around the left second premolar, with a fistula leading into the maxillary sinus. The partial dentition of this elderly man also has large calculus deposits. Periodontal disease and antemortem tooth loss are rare, although poor preservation of jaws and alveolar tissue made these processes difficult to study. By contrast, enamel hypoplasias are seen in 68% of individuals (*n* = 13/19 dentitions that could be scored). Defects include transverse lines (linear enamel hypoplasia, or LEH), grooves, and pits.

The crania of only five of 49 individuals could be assessed for cribra orbitalia and porotic hyperostosis. An adult woman (GeJi9 B-24A) presents the sole case, with cribriform lesions visible on the orbital roof paired with minor periosteal activity on the right supraorbital area. Another individual, an elderly male over 50 years old (GeJi10 B-3), has indications of low bone mass suggestive of osteoporosis.

Cases of possible infectious disease include GeJi9 B-7, an infant aged 6 to 12 months whose frontal bone shows disorganized new bone endocranially, on the squamous portion. The endocranial bone is highly thickened and vascularized but the outer table appears normal, consistent with a non-specific indicator of haemorrhage or infection (Lewis 2004). GeJi9 B-21A (male, 20–30 years) and GeJi9 B-28 (female, 30–40 years) both show non-taphonomic erosion around the pubic symphysis from either infection or pelvic stress injury. We also documented healed fractures on various fingers, toes, and ribs on three individuals (GeJi9 B-1, B-2, B-25). Healed fractures may also account for mild OA on two rib heads of GcJh5 B-1 and a discrepancy in femur lengths in GeJi9 B-6A.

The young woman from Manemanya buried with thousands of beads (GcJh5 B-1) shows several skeletal anomalies that suggest asymmetries to her face and jaw. Her left zygomatic bears a bony spur along the temporal margin where the temporal fascia attach. This feature, which is not present on the well-preserved right side, may be related to the pattern of greater wear on the right side of the dentition, greater dental calculus on the left side, and asymmetry in the mandibular rami. The right mandibular ramus is more prominently inverted at gonion, with asymmetries extending through the submandibular fossa region (inferior to the mylohyoid line). Other distinguishing features of her skull include four large wormian bones and a notably thin occipital squamous portion which, taken in concert, may suggest a congenital or developmental origin for the asymmetry. Facial asymmetry can arise from congenital, developmental, and acquired factors (Thiesen et al. 2015). One possibility in this case is congenital muscular torticollis (wry-neck). Limited ranges of motion and repose can lead to asymmetries of the face and head (Nilesh and Mukherji 2013). Given that she was also relatively tall (~ 170-180 cm, based on estimated maximum femur length, measured in situ), perhaps her appearance and bearing were in some ways linked to her burial treatment.

### Biomechanical indicators of mobility

**Fig. 8:**
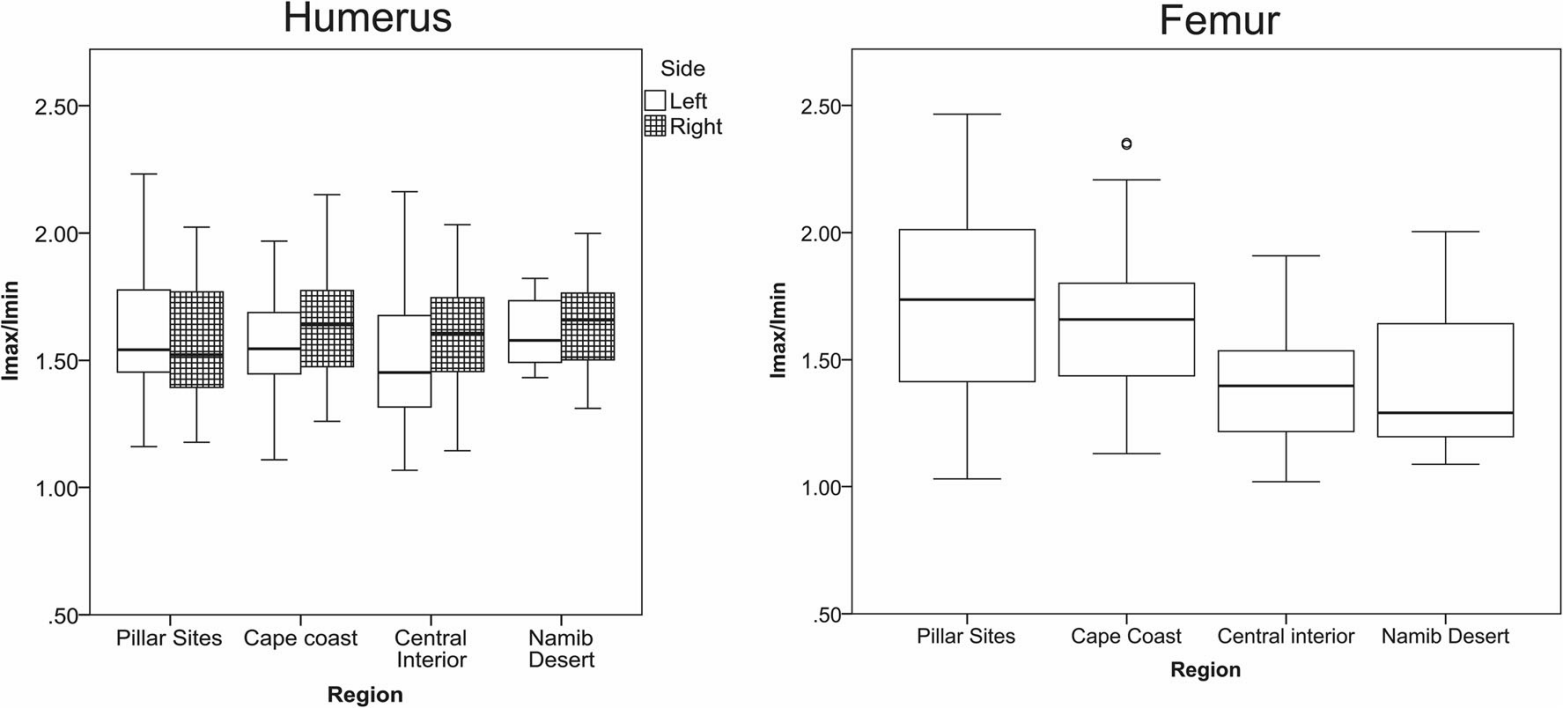
CSG *I*_max_/*I*_min_ ratios for humerus and femur midshafts for the pillar site sample compared to southern African Later Stone Age Cape Coastal, Central Interior, and Namib Desert foragers and herders

Biomechanical measures obtained from the 34 eligible midshaft elements are presented in Table 5. Table 6 contains mean values for elements analysed as well as summary data for the southern African comparative groups. For all skeletal elements, *I*_max_/*I*_min_ values fall within the range of variation of the southern African herding and foraging groups (examples in Fig. 8). There are no significant differences in *I*_max_/*I*_min_ for any of the upper limb elements. The *I*_max_/*I*_min_ values for the pillar site tibia and femora recovered are comparatively high relative to the southern African groups; however, tibia CSG property data are very limited. For the femora, there are significant differences between the four groups considered (*p* = 0.00). However, there are no pairwise significant differences, likely due to the small sample sizes.

**Table 5:** Cross-sectional geometric (CSG) properties for pillar site individuals

Skeleton	Est. Sex	Side	I_max_/I_min_
HUMERUS			
GcJh5 B-1	F	L	1.16
GcJh5 B-1	F	R	1.49
GeJi9 B-6A	M	R	1.55
GeJi9 B-6B	?	–	1.53
GeJi9 B-3	M?	L	2.23
GeJi9 B-3	M?	R	1.77
GeJi9 B-9	F	L	1.78
GeJi9 B-11	F	L	1.45
GeJi9 B-18B	M	R	2.02
GeJi9 B-20	F?	R	1.39
FEMUR			
GcJh5 B-1	F	R	1.44
GeJi9 B-6A"	M	L	2.21
GeJi9 B-6A"	M	R	1.75
GeJi9 B-9	F	R	1.39
GeJi9 B-11	F	R	1.72
GeJI9 B-12	M	R	1.03
GeJi10 B-3	M	R	2.47
GbJj1 B-1	M	R	1.81
TIBIA			
GeJi9 B-6A	M	R	2.61
CLAVICLE			
GeJi9 B-3	M?	L	1.49
GeJi9 B-3	M?	R	1.39
GeJi9 B-9	F	L	1.54
GeJi9 B15A	F?	R	2.11
GeJi9 B-18B	M	L	2.04
GeJi9 B-20	F?	L	1.60
GeJi9 B-26A	M	L	2.13
GeJi9 B-26A	?	R	1.45
ULNA			
GeJi9 B-3	M?	L	1.58
GeJi9 B-9	F	L	1.68
GeJi9 B-18B	M	R	1.15
GeJi9 B-30	M	L	1.27
RADIUS			
GeJi9 B-9	F	L	1.48
GeJi9 B-9	F	R	1.41
GeJi9 B-18B	M	R	1.46

**Table 6:** Results of Kruskal-Willis ANOVAs comparing *I*_max_/*I*_min_ CSG properties

Element	Sample	*n*	Mean	S.D.	*p* value
Left humerus	Cape coast	75	1.56	0.20	
	Central interior	36	1.52	0.26	
	Namib Desert	10	1.59	0.20	
	Pillar Sites	5	1.63	0.40	
					0.489
Right humerus	Cape coast	67	1.65	0.21	
	Central interior	32	1.51	0.23	
	Namib Desert	12	1.64	0.20	
	Pillar Sites	6	1.57	0.30	
					0.764
Femora	Cape coast	66	1.65	0.27	
	Central interior	47	1.41	0.24	
	Namib Desert	17	1.40	0.27	
	Pillar Sites	8	1.73	0.46	
					0.000
Tibia	Cape coast	44	2.46	0.44	
	Central interior	43	2.26	0.40	
	Namib Desert	16	2.15	0.39	
	Pillar Sites	1	2.61		
					0.084
Left clavicle	Cape coast	36	1.95	0.33	
	Pillar Sites	5	1.76	0.30	
					0.162
Right clavicle	Cape coast	41	1.85	0.40	
	Pillar Sites	3	1.65	0.40	
					0.321
Left ulna	Cape coast	34	1.39	0.25	
	Pillar Sites	3	1.51	0.22	
					0.293
Right ulna	Cape coast	32	1.36	0.22	
	Pillar Sites	1	2.04		
Left radius	Cape coast	36	1.41	0.19	
	Pillar Sites	1	1.48		
					0.757
Right radius	Cape coast	33	1.51	0.22	
	Pillar Sites	2	1.43	0.04	
					0.659

## Discussion

Bioarchaeological insights from the western pillar sites offer fresh perspectives on the construction and use of pillar sites, as well as novel insights into the lived experienced of those buried there. These results advance our knowledge on three distinct fronts: mortuary behaviours at these sites, patterns of inter-site variability, and skeletal indicators of health, disease, and mobility among eastern Africa’s first herders.

### Mortuary patterns at the pillar sites

Excavations at the four pillar sites west of Lake Turkana lend additional support for the hypothesis that the pillar sites functioned as cemeteries. Building on previous identifications of human remains at Lothagam North and Manemanya, our work has now confirmed the presence of burials in the central platform at Lothagam West, and in potentially younger cairn features at Lothagam West and Kalokol. The presence of a mortuary cavity in Kalokol’s platform, beneath a centrally positioned acacia tree, seems possible and may be tested with additional excavations.

The ‘Jarigole Mortuary Tradition’ can now be revised. Although secondary bundle burials were present within platform deposits at Lothagam North, most interments were primary, as were the few burials excavated at Lothagam West and Manemanya. ‘Clothed’ bodies, at least with adornments, had been prepared for individual placement. At Lothagam North, both primary and secondary burials were present as discrete features and are likely to have been the source of the isolated fragments found between the bodies within the cobble-filled matrix. None of the western site platforms resemble ossuaries strictly intended for secondary remains, and we did not observe the degree of commingling reported at Jarigole (Nelson 1995). Most human remains recovered from Jarigole are fragments and/or small, mobile elements such as teeth (personal observation, National Museums of Kenya), similar to those observed in the ~ 50 cm upper cap deposits at Lothagam North. Future excavations at Jarigole targeting deeper deposits would test the hypothesis that primary burials exist in deeper strata. We do not know whether the architecture of the mortuary cavity and density of burials at Lothagam North are typical. Although multiple burials were encountered at Lothagam West and Manemanya, neither site presented the deeply stratified mortuary deposits observed at Lothagam North (Fig. 5).

Based on available evidence, pillar sites are cemeteries, although the extent to which this was their primary function remains unclear. Most of the burials are primary, indicating that people were brought to the site shortly after death, contradicting previous notions. This fact raises additional questions about mobility, social organization, and funerary practices among groups who used these sites. Was each pillar site used by a specific group and/or extended family who either lived nearby or travelled great distances immediately after a death? Or were individuals interred based on when and where they died or some combination of other factors? Why were some people included as secondary burials? And did funerals trigger large community gatherings or were they smaller affairs carried out by a subset of relatives? To answer these questions, we need more information about who was buried at specific pillar sites and in what contexts. As research to date has focused largely on Lothagam North, future excavations should target other sites, particularly Kalokol and Manemanya, to probe patterns of mortuary variation.

### Intra-site variability: personal adornment

Although material culture varies among the western pillar sites (Grillo and Hildebrand 2013; Hildebrand et al. 2011), bioarchaeological excavations add another layer of complexity: items directly associated with buried people. With a few potential exceptions, such as the bone and ivory object at Manemanya and the zoomorphic palette at Lothagam North, all examples appear to be adornments rather than burial gifts. At Lothagam North, stone beads were associated with most primary burials in the central platform in diverse combinations of number, raw material, and colour (Figs. 5 and 6, Table 2). Although some of the stones may have originated nearby, others would have come from more distant and distinct sources. Beads were found with men, women, and children, and they varied in pattern from person to person. Burials that could not be associated with any ornamentation were also the most incomplete and poorly preserved, so may have been associated with other beads found in the platform fill. The ubiquity of beads with the burials suggests that interment with adornment was the norm. Patterns of bead distribution, combined with the presence of men, women, and children within the central platform, suggests minimal social stratification among people buried there (Hildebrand et al. 2018).

The elaborate burial encountered at Manemanya (GcJh5 B-1) may reflect unique circumstances. The unidentified items lain to her right and the strands of beads strewn on her body after interment distinguish this burial from those at Lothagam North, where almost all adornments could have been worn by the deceased. The hundreds of stone beads and thousands of OES beads covering her body may signify special status or identity within the group. Stone bead raw materials are quite different from those observed at Lothagam North and Jarigole. Calcite and sandstone that would have been readily available from local sources are dominant at Manemanya. Other ornaments, such as the perforated lion tooth pendant, were likely difficult to procure, suggesting her bead choices may have been stylistic rather than economic. Future excavations at Manemanya may reveal whether this burial’s degree and types of ornamentation were exceptional or the norm.

Bead and other burial inclusion differences between Lothagam North and Manemanya, and between these sites and Lothagam West and Kalokol (where few to no beads were recovered) are topics that merit further investigation. Grillo and Hildebrand (2013) note that differences between pillar sites do not easily track with geography or time; Lothagam North and Jarigole are the most artifactually similar despite their positions on opposite sides of the lake (~ 300 km apart by land and ~ 100 km by water), while Lothagam North and West are quite different despite close proximity and evidence of use within the same 60-year period. Because it can be assumed that the people using the two contemporaneous pillar sites on Lothagam were aware of each other, differences in material culture must reflect some other aspect of function, identity, or preference. That said, some aspects of this tradition could well have shifted over the centuries-long span of pillar site activity, and later burial events may reflect very different dynamics and social organisation those from earlier times.

### Skeletal indicators for health, disease, and activity

This new skeletal sample represents one of the largest collections from across eastern Africa, and the only one known to represent the region’s earliest food producers. Bioarchaeological datasets provide a first glimpse into who was buried at the pillar sites and their lived experiences. Burials at Lothagam North seemingly represent a cross-section of a community in terms of age and sex, suggesting most members of the group may have been buried there. While excavations at the other sites have been more minimal, the cairn burial of a child at Lothagam West and heavily ornamented young woman at Manemanya raise other questions about early herder social organization. Circumstances leading to burial within a pillar site remain an ongoing topic of investigation.

Skeletal analyses provide several insights into the lives of these individuals. Prevalent conditions like OA suggest that people were living many years into adulthood, and evidence for more serious diseases and/or traumatic injuries is rare. However, systematic assessments were limited by the fragmentary nature of the remains, particularly the crania which were often crushed by a heavy boulder or slab. Dental health was relatively good with slight wear and low rates of caries, but a high prevalence of dental calculus. A non-cariogenic diet low in carbohydrates seems to have been more influential in deterring carious lesions than rapid tooth wear or cleaning (for which there is no evidence). Most individuals exhibit enamel hypoplasias suggesting some health stressors during development. LEH rates are likely underestimates as the teeth were too fragile to cast for microscopic study; future work will require a novel approach.

Limb CSG properties fall within the range of southern African foragers whose subsistence patterns required them to move frequently across the landscape. Upper limb *I*_max_*/*I_min_ values suggest that early herder lifeways around Lake Turkana required similar types of limb activities as those used by prehistoric southern African populations, although this cannot be associated with a specific activity or movement type. Southern African forager upper limb CSG properties differ from those of contemporary swimmers (Shaw and Stock 2013), as well as from archaeological groups who engaged in watercraft use (Stock and Pfeiffer 2001). Comparable upper limb morphologies suggest that the people at the pillar sites around Lake Turkana likely did not engage in heavy use of watercraft (i.e. paddling) or frequent bouts of vigorous swimming to obtain freshwater resources.

There are significant differences between the femur *I*_max_/*I*_min_ values among the four groups, although no pairwise differences reach significance. Pillar site femur *I*_max_/*I*_min_ values are higher than Central Interior and Namib Desert values, and most similar to those of Cape Coast foragers (Fig. 8). Greater lower limb *I*_max_/*I*_min_ values have been associated with mobility across complex (but not necessarily high relief) terrain (Higgins 2014), as well as unidirectional lower limb movement, such as those undertaken by marathon runners (Shaw and Stock 2009). Although both factors are known to produce high *I*_max_/*I*_min_ ratios, it is not possible to determine which factor was the primary driver in archaeological samples. Among the Cape Coast foragers, high values are attributed to an intensive mobile foraging lifestyle in a region with complex terrain, including rocky shorelines (Stock and Pfeiffer 2004; Cameron and Stock 2018). Slightly lower values among Coastal Interior and Namib Desert groups likely reflect high mobility but on less-complex landscapes (Cameron and Stock 2018). Similarities between the pillar site sample and Cape Coast foragers can be interpreted in two ways: either early herders around Lake Turkana moved across comparably complex (but not necessarily similar) terrain or their terrestrial mobility patterns were sufficiently intense to produce an equivalently high signal. These possibilities provide a starting point for further study of mid-Holocene Turkana landscapes and how people were moving across them. However, interpretation is limited by the small number of skeletons that were complete enough to assess for CSG properties, as well as the lack of eastern African comparative samples.

It is difficult to contextualize those buried at the pillar sites among other human populations because we know little about the health of ancient herders or forager societies transitioning to mobile pastoralism. Research on contemporary African pastoralists facing sedentarisation suggests that nomadic groups maintain lower levels of malnutrition and morbidity than settled groups, although this is complicated by other factors such as access to medical care (Fratkin and Abella Roth 2005). Some attributes of the western pillar site sample, such as their low rates of dental caries and infectious disease, may indicate similar diet and health patterns between ancient and contemporary mobile pastoralists in eastern Africa. Skeletal evidence consistent with high mobility and/or mobility across complex terrain suggests other potential parallels. Ongoing bioarchaeological work at the pillar sites, as well as fisher-forager sites in Turkana such as Lothagam Lokam (Goldstein et al. 2017), may provide additional insights into the biocultural impacts of the transition to herding as well as permit comparisons of health and behaviour between past vs. present-day pastoralists.

## Conclusions

Bioarchaeological investigations at four megalithic pillar sites west of Lake Turkana have revealed important information on the behaviour and biology of the first herders in eastern Africa. Our findings confirm some previous assumptions about this mortuary tradition and challenge others. All pillar sites do appear to have mortuary components, and at least one (Lothagam North) likely accommodated hundreds of people. However, most burials on the west side of Lake Turkana are primary. This indicates that people were usually buried shortly after death rather than receiving prior treatment and subsequent inclusion as secondary burials (although both types of burial are present at Lothagam North). Further work is needed to discern whether primary burials predominate in deeper deposits at pillar sites on the east side of the lake, and the extent of mortuary deposits at Lothagam West, Manemanya, and Kalokol.

Stone and OES beads recovered from Lothagam North are similar to those previously excavated from Jarigole, but at Lothagam North, their function as adornments within primary interments is clear. The presence of beads with most burials, without apparent patterning regarding age and sex, suggests the absence of strong social hierarchies (Hildebrand et al. 2018). However, an elaborate burial excavated from Manemanya with thousands of beads, many of types distinct from those at Lothagam North, raises additional questions about material cultural variability among pillar sites and the complexity of social roles within these communities. As pillar sites represent a globally rare example of monumentality among mobile herders in unpredictable circumstances, beads and other artefacts provide important clues about the social processes and institutions that helped people manage risk.

Beyond mortuary and material culture insights, this work has yielded a unique collection of archaeological human remains from an early food-producing society in Africa. Little is known about human populations from this region and time period, or about groups transitioning from fishing and foraging to mobile herding. Regional consequences of the shift to food production have long been a topic of bioarchaeological inquiry, but few studies have focused on early pastoralism given a lack of suitable skeletal samples in Africa and beyond. Human remains from the pillar sites provide a record of people who lived through major environmental and economic shifts, permitting us to explore their dynamic social responses. Not only do monumental cemeteries around Lake Turkana capture a key time period in eastern Africa, they offer important perspectives on how humans react and adapt to significant changes in the world around them.

## Footnotes

1 Note: Several versions of Nelson 1995 are in circulation: (a) the original print version of the Kenya Past & Present article, (b) a PDF version, (c) HTML versions of which one is currently available at http://www.chaz.org/Arch/Turkana/Jarigole/Jarigole.htm, and (d) a Word version. Not all versions of (b), (c), and (d) downloaded or exchanged between colleagues over the years have the entirety of information presented in (a). We are using (a), the original print version.
